# Design and motion control of exoskeleton robot for paralyzed lower limb rehabilitation

**DOI:** 10.3389/fnins.2024.1355052

**Published:** 2024-02-21

**Authors:** Zhiyong Zhu, Lingyan Liu, Wenbin Zhang, Cong Jiang, Xingsong Wang, Jie Li

**Affiliations:** ^1^College of Automation, Nanjing University of Posts and Telecommunications, Nanjing, China; ^2^School of Mechanical Engineering, Southeast University, Nanjing, China; ^3^College of Computer Science and Software Engineering, Hohai University, Nanjing, Jiangsu, China

**Keywords:** exoskeleton robots, behavior-assistant robots, human-robot systems, motion control, rehabilitation application

## Abstract

**Introduction:**

Patients suffering from limb movement disorders require more complete rehabilitation treatment, and there is a huge demand for rehabilitation exoskeleton robots. Flexible and reliable motion control of exoskeleton robots is very important for patient rehabilitation.

**Methods:**

This paper proposes a novel exoskeleton robotic system for lower limb rehabilitation. The designed lower limb rehabilitation exoskeleton robot mechanism is mainly composed of the hip joint mechanism, the knee joint mechanism and the ankle joint mechanism. The forces and motion of the exoskeleton robot were analyzed in detail to determine its design parameters. The robot control system was developed to implement closed-loop position control and trajectory planning control of each joint mechanism.

**Results:**

Multiple experiments and tests were carried out to verify robot's performance and practicality. In the robot angular response experiments, the joint mechanism could quickly adjust to different desired angles, including 15°, 30°, 45°, and 60°. In the trajectory tracking experiments, the exoskeleton robot could complete tracking movements of typical actions such as walking, standing up, sitting down, go upstairs and go downstairs, with a maximum tracking error of ±5°. Robotic wearing tests on normal people were performed to verify the assistive effects of the lower limb rehabilitation exoskeleton at different stages.

**Discussion:**

The experimental results indicated that the exoskeleton robot has excellent reliability and practicality. The application of this exoskeleton robotic system will help paralyzed patients perform some daily movements and sports.

## 1 Introduction

The research direction of lower limb rehabilitation exoskeletons is focused on the design of these devices for patients with paraplegia, an area that is grounded in bionics principles and informed by a multidisciplinary intersection of mechanical engineering, electrical engineering, biomedical sciences, human bionics, artificial intelligence, and sensing technologies (Plaza et al., 2023). Through the strategic integration of various sensors, a diverse array of technologies spanning sensing, signal acquisition, and microcomputing were harnessed to inform the design of these rehabilitation robotic systems (Sarajchi et al., 2021).

In the context of paraplegia patient care, traditional rehabilitation therapies typically involve one-on-one or multiple-therapist-to-one treatment modalities administered by rehabilitation therapists (Wang et al., 2022). However, these approaches are often characterized by inefficiencies, difficulties in movement control and effect assurance, challenges in rehabilitation assessment, and a shortage of qualified healthcare professionals (Manuli et al., 2021). Rehabilitation exoskeletons offer a compelling alternative in this scenario, as they can significantly reduce the workload burdening rehabilitation departments, effectively liberating these departments and enhancing treatment efficiencies. Moreover, these exoskeletons have the potential to promote patient engagement in rehabilitation training, while also enabling objective evaluations of training intensity, duration, and outcomes. Consequently, patients can benefit from more systematic, comprehensive, and standardized rehabilitation interventions (Pinto-Fernandez et al., 2020).

For individuals with lower limb injuries, the utilization of lower limb rehabilitation exoskeletons can play a pivotal role in facilitating normal daily activities. These exoskeletons not only address various challenges related to medical resource allocation and manual training in rehabilitation settings, but also allow for precise measurements of human kinematics and physiological data through sophisticated sensory systems. These measurements allow rehabilitation physicians to more accurately assess the patient's condition (de Miguel-Fernández et al., 2023) and provide an objective foundation for refining and optimizing rehabilitation programs tailored to individual patient needs (Plaza et al., 2021; Su et al., 2023).

With the enhancement of people's living standards, individuals afflicted with limb movement disorders will increasingly pursue more comprehensive rehabilitation therapies. Consequently, the demand for these rehabilitative treatments will continue to escalate. The lower limb rehabilitation exoskeleton, a specialized medical device designed for patients with lower limb paralysis or disabilities, will occupy a pivotal position in rehabilitation therapies (Huamanchahua et al., 2021). The rehabilitation exoskeleton robot is an industrialization research topic with significant market prospects. The development of rehabilitation robots also plays an important role in the technological development of medical rehabilitation.

Currently, exoskeletons for paraplegic patients are divided into two main categories: rehabilitation exoskeleton robots consisting of an exoskeleton, crutches or auxiliary support mechanisms, and a control handle, such as the ReWalk rehabilitation^1^ (Zeilig et al., 2012) bionic robotic leg (Esquenazi et al., 2017); and exoskeletons that do not need crutches or other auxiliary support mechanisms^2^ (Esquenazi et al., 2012). Trajectory tracking control (Aole et al., 2020) is the main control method in most of the current exoskeleton robots (Andrade et al., 2021) and plays an important role in the operation and implementation of exoskeleton robots (Shi et al., 2019; Li et al., 2021). Traditional robotic arm modeling and control theories have laid an important foundation for the modeling, analysis and control of lower limb exoskeleton robots (Caulcrick et al., 2021; Shi et al., 2021), such as the sensitivity amplification control (Zheng, 2021), identification of the dynamic model (Bryan et al., 2021) and real-time adjustment of torque.

Most current research predominantly concentrates on walking states, it doesn't adequately account for the diverse daily life scenarios encountered by paraplegic patients, such as activities like standing up, sitting down, navigating stairs, or managing slight inclines. Moreover, the trajectory tracking control methodologies commonly utilized often rely on trajectories derived from the movements of able-bodied individuals in their daily routines, overlooking the unique circumstances and needs of patients who use crutches (Embry and Gregg, 2020).

In this paper, a multi-scenario and full-process rehabilitation exoskeleton robot system for paraplegic patients is proposed, which can realize daily actions such as walking, standing and sitting. The designed exoskeleton robot contains active hip joint mechanism, active knee joint mechanism and passive ankle joint mechanism. Detailed mechanical analysis and design were performed for the exoskeleton joint mechanisms. The exoskeleton control system combined sensors and drive motors could achieve closed-loop control and tracking motion of each exoskeleton joint. Motion response experiments and robot trajectory tracking experiments were conducted to verify its response performance and reliability. Multiple groups of normal people wore exoskeletons to test the assistance effect of walking, standing up, sitting down and other movements. Series of experiments and tests verified the practicability and stability of the lower limb rehabilitation exoskeleton robot. This exoskeleton robot system can help paraplegic patients recover and greatly enhance their mobility.

## 2 Exoskeleton robot design and analysis

### 2.1 Robot principle

Human lower limb movement represents a sophisticated and systematic process, initiated by the brain's dissemination of intentional movement information (Leech et al., 2022). This information is conveyed through nerve conduction via the spinal cord, extending to the nerves innervating the lower limbs. Subsequently, these nerves exercise control over the contraction and extension of lower limb muscles, which ultimately impetus the rotational movement of skeletal joints. Although it is possible for exoskeleton robot active joints to generate greater torque than human joints, the number and degrees of freedom (DOF) of robot joints are typically much lower than the corresponding number in the human body. This disparity necessitates the orchestration of coordinated movement between the human body, characterized by an extensive range of DOF, and the exoskeleton, which operates with a more restricted range. The motion control algorithms employed in exoskeletons should be meticulously designed to accommodate the inherent joint motion characteristics of the human body, with the aim of minimizing the sense of discomfort and discomfiture during human-machine interaction.

**Figure 1:**
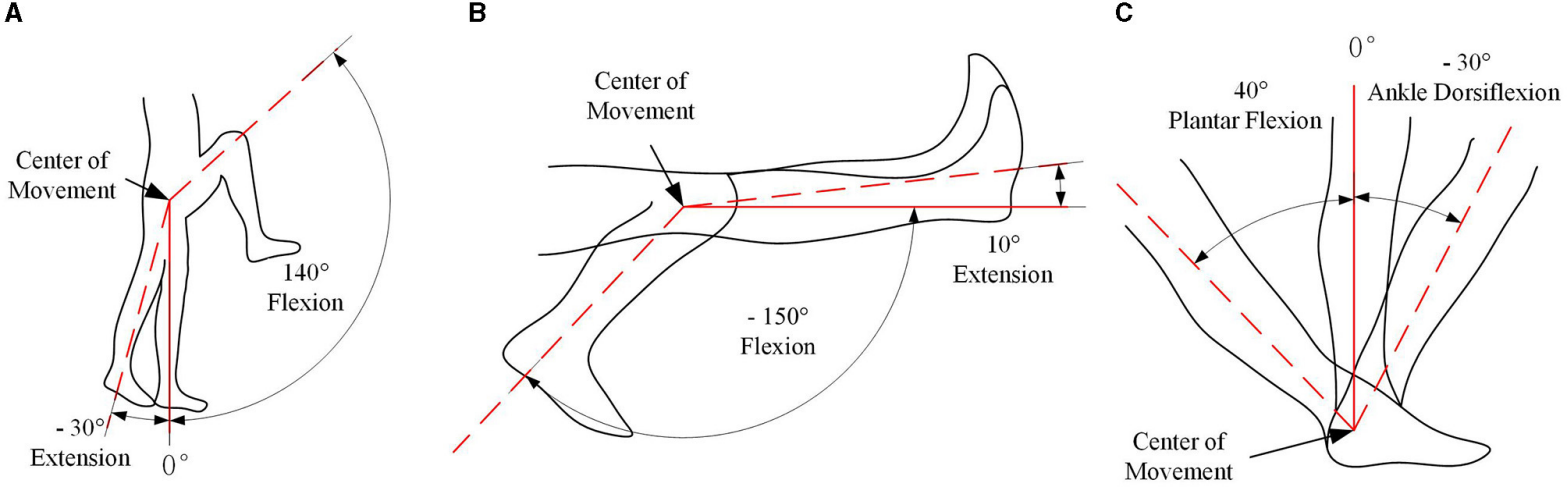
Movement mechanism diagram of the main joints of the human lower limbs. **(A)** Hip joint movement; **(B)** knee joint movement; **(C)** ankle joint movement.

The human lower limb comprises three primary joints, namely the hip, knee, and ankle joints, which collectively facilitate a wide range of locomotive functions. The main joint movement mechanisms of the human lower limbs are presented in Figure 1. Considering the particularity of the exoskeleton robot being applied to patients with lower limb paralysis, in order to achieve walking purposes, the exoskeleton robot does not need to have the motion performance of all joints. Additionally, given the practical constraints posed by the need to optimize the size and weight of the exoskeleton, a judicious selection of joints is warranted. The designed lower limb exoskeleton robot has a total of 10 DOFs across both legs, with the hip and knee joints, each endowed with a single DOF, serving as active drivers of motion. Simultaneously, the ankle joint, endowed with three DOFs, operates as a passive joint. The DOF number of the lower lime exoskeleton robot is presented in Table 1.

**Table 1:** The lower limb DOF of the human and the developed exoskeleton robot.

**DOF**	**Hip**	**Knee**	**Ankle**
Human lower limb	3	1	3
Lower limb exoskeleton robot	1	1	3

In consideration of the operational context of wearable lower limb exoskeletons, each joint motion range should be consistent with the normal pedestrian walking. However, prioritizing the safety and wellbeing of the wearer, it is judicious to design the motion range of the exoskeleton system to be slightly restricted compared to that of human joints. The detail joint motion range on sagittal plane is presented in Table 2.

**Table 2:** Joint motion range of human and exoskeleton on sagittal plane.

**Joint**	**Human motion range**	**Exoskeleton motion range**
Hip flexion/extension	−30° to 140°	−30° to 115°
Knee flexion/extension	−150° to 10°	−100° to 0°
Ankle dorsiflexion/plantar flexion	−30° to 40°	−20° to 20°

### 2.2 Robot mechanical design

The lower limb rehabilitation exoskeleton is a wearable mechanical system designed for patients with lower limb paralysis. This system integrates robotics technology, automation control theory, and clinical medical technology, culminating in an automated robotic device dedicated to facilitating a wide range of daily activities for these patients. When designing this robot system, the following critical factors should be considered:

(1) Rational allocation of DOF: given the exoskeleton's primary function of assisting paralyzed patients in accomplishing daily tasks—such as standing, sitting, climbing stairs, and walking—it is paramount to precisely determine joint positions and DOF. This ensures optimal support for these activities while mitigating the risk of secondary injuries to the patient;
(2) Adjustability of the mechanism: the application of the lower limb rehabilitation exoskeleton encompasses a vast age range, significant height disparities, and diverse body types. Consequently, the design process must accommodate patients with varying heights and weights by incorporating adjustable features such as leg bar length, waist width, and strap mechanisms;
(3) Reasonable allocation of joint movement range: while ensuring the basic range of motion of each joint, it is imperative to anticipate extreme scenarios, such as drive failures. To mitigate potential risks, safety limits must be designed to distribute joint movement ranges in a manner that guarantees wearer safety and prevents secondary injuries;
(4) Convenient wearability: as the lower limb rehabilitation exoskeleton is worn externally on the human body, it must prioritize wearability. Ideally, after a concise user training, individuals should be able to effortlessly don and doff the exoskeleton. This requires a thoughtful design approach that balances complexity with usability, ensuring maximum comfort and ease of use for the wearer.

**Figure 2:**
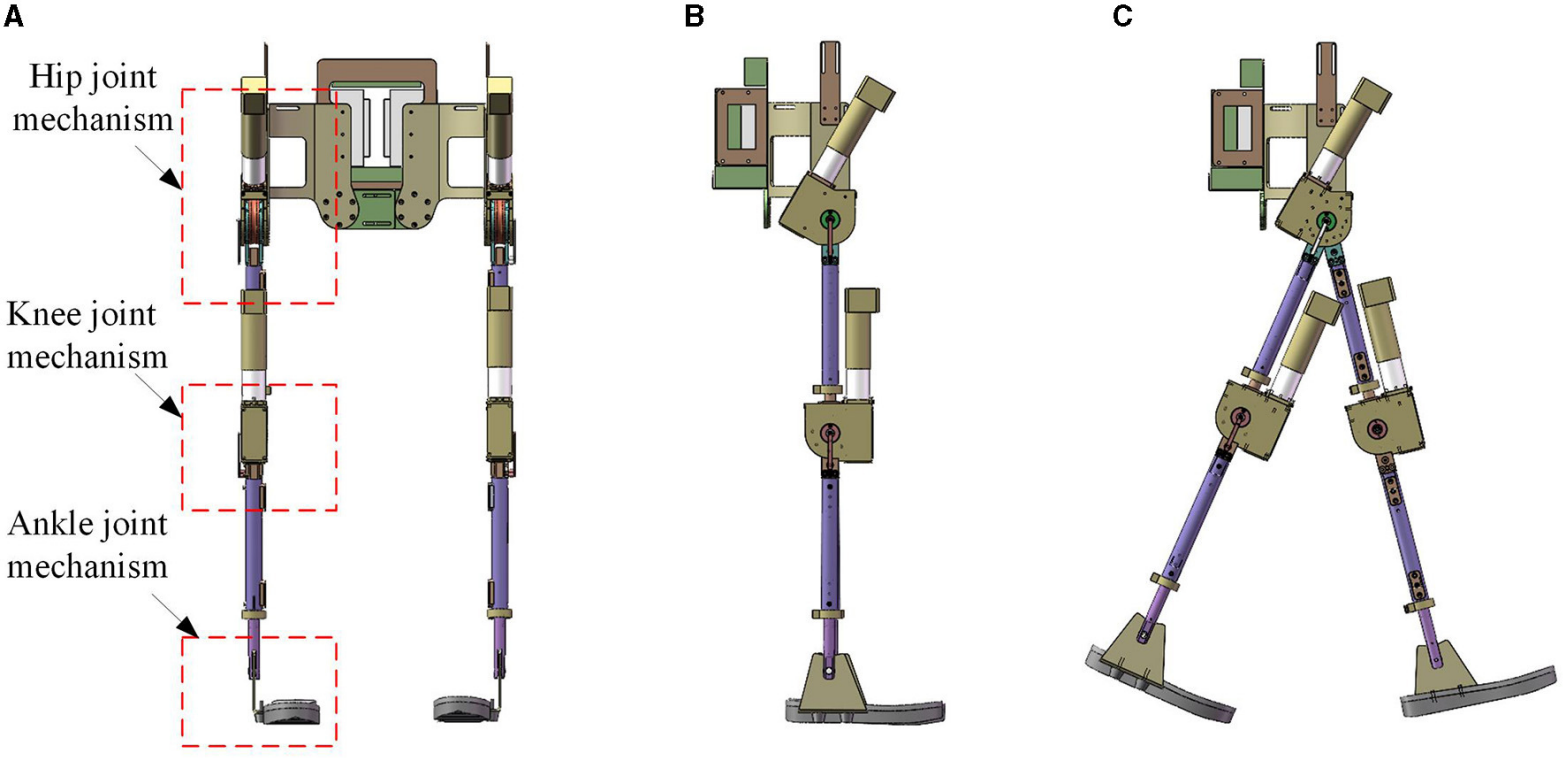
Design of lower limb rehabilitation exoskeleton. **(A)** Front view; **(B)** side view of upright state; **(C)** side view of stepping state.

According to the above design points, as shown in Figure 2, a complete set of lower limb rehabilitation exoskeleton robot is proposed. The overall weight of this exoskeleton robot is <20 Kg. The designed exoskeleton robot mainly includes the backpack mechanism, the hip joint mechanism, the knee joint mechanism and the ankle joint mechanism.

**Figure 3:**
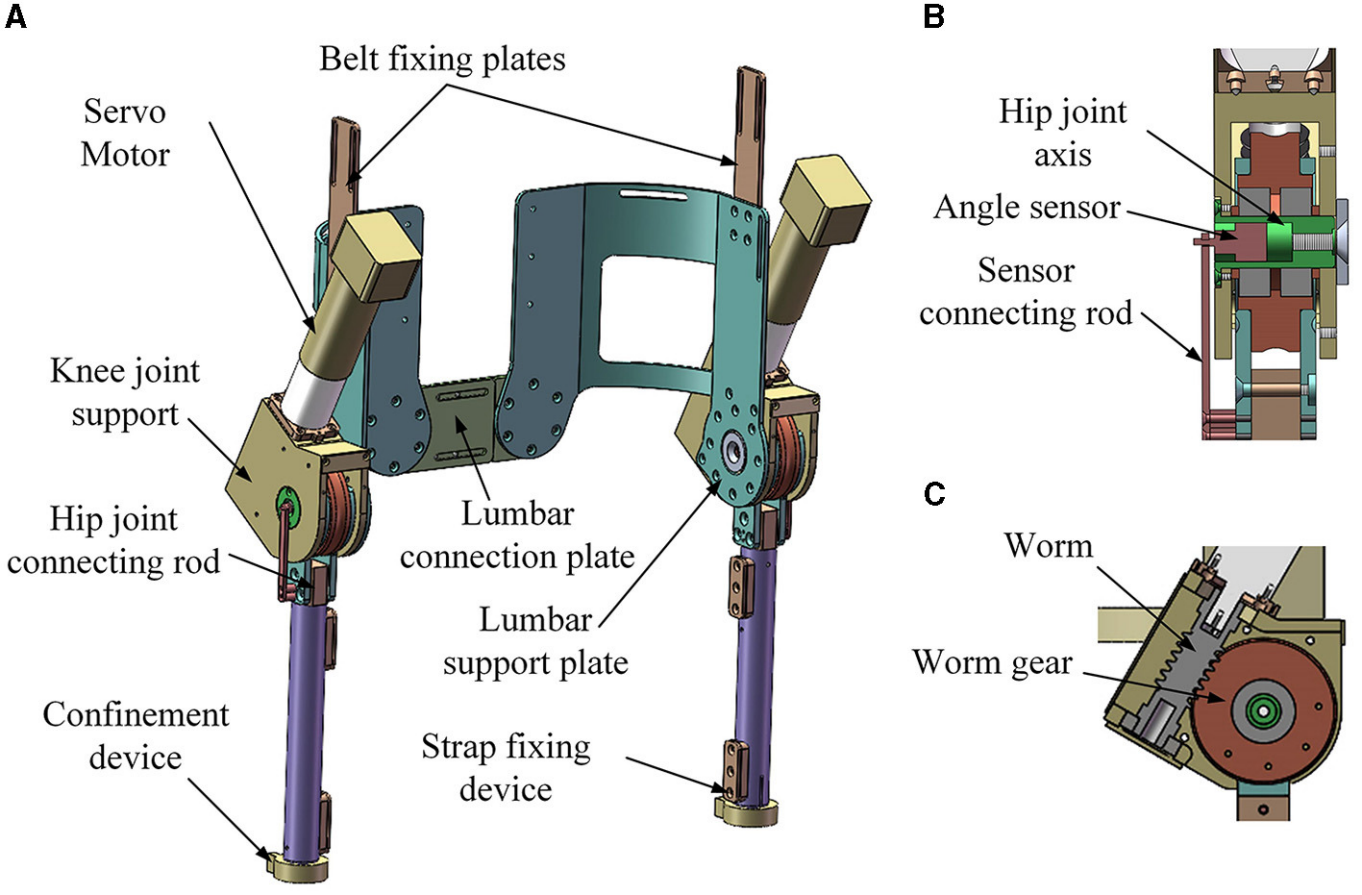
Hip joint mechanism of the lower limb rehabilitation exoskeleton. **(A)** Overall view; **(B)** front section view; **(C)** side section view.

The hip joint of the lower limb rehabilitation exoskeleton holds paramount importance, given its integral involvement in the majority of movements during daily exercises. Taking into account the prerequisites of safety, reliability, and practicality pertinent to patients with lower limb paralysis, the hip joint is designed as a driven joint. Figure 3 shows the hip joint mechanism of the lower limb rehabilitation exoskeleton.

Two hip joint mechanisms of the lower limb rehabilitation exoskeleton robot contain the following main components: servo motors, worms, worm gears, hip joint connecting rods, thigh poles, hip joint supports, hip joint axes, lumbar support plates, belt fixing plates, strap fixing devices, safety limit devices, confinement devices, sensor devices and a lumbar connecting plate. The sensor device contains sensor and sensor connecting rod.

The transmission process unfolds as follows: the servo motor initiates the motion by driving the reducer, which subsequently propels the worm. The worm then engages the worm gear, leading to the rotation of the hip joint connecting rod. This rotation translates into the motion of the thigh pole, facilitating thigh movements in the human body. Concurrently, the sensor device plays a pivotal role by measuring the hip joint angle for purposes of real-time control and precision.

**Figure 4:**
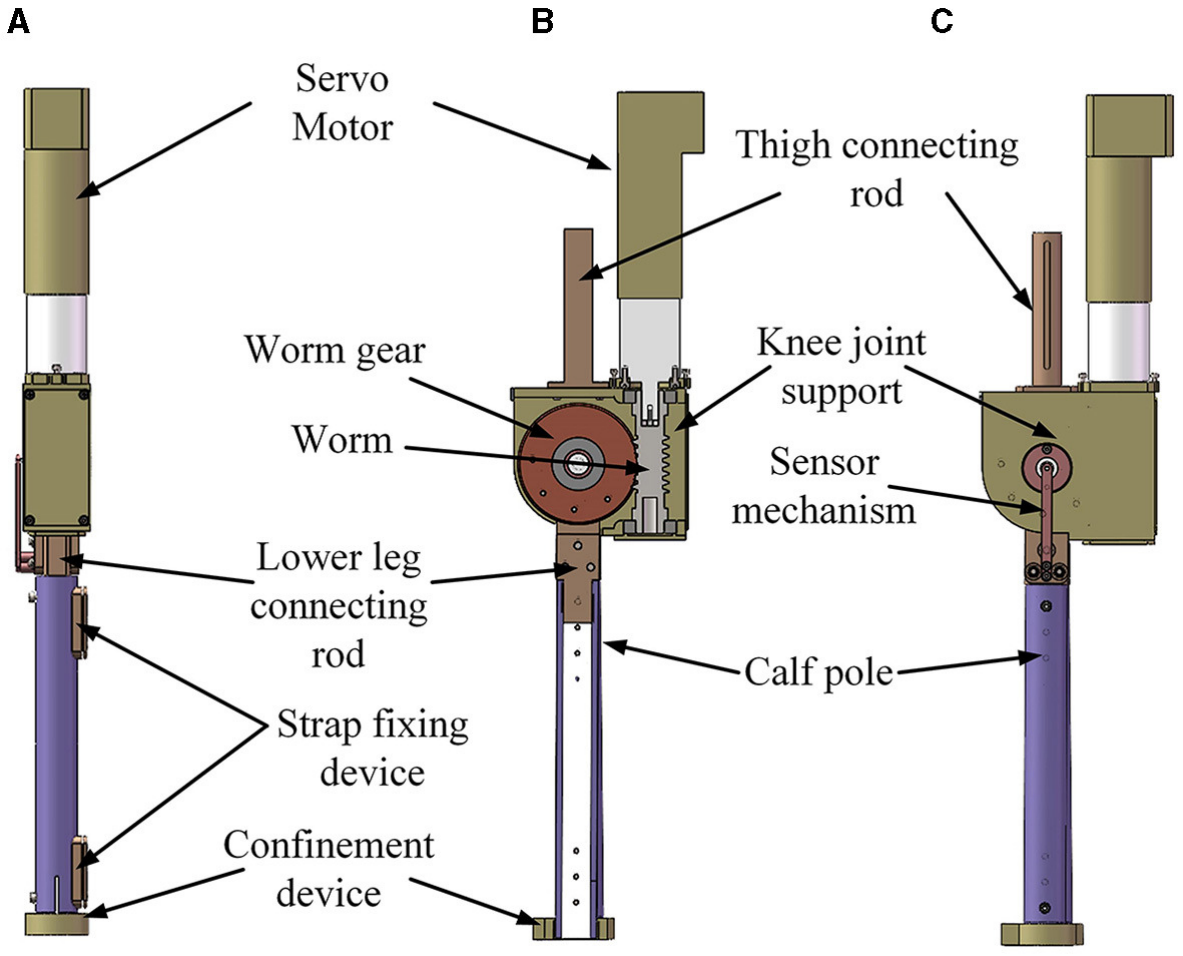
Knee joint mechanism of lower limb rehabilitation exoskeleton. **(A)** Front view; **(B)** section view; **(C)** side view.

In the daily movements executed by the human body, the involvement of the knee joint is almost ubiquitous, underscoring its importance in the lower limb rehabilitation exoskeleton. The knee joint mechanism of the lower limb rehabilitation exoskeleton is designed as an active joint. It also uses the worm gear transmission method of the hip joint mechanism, and its core structure is similar to that of the hip joint mechanism. The knee joint mechanism of the lower limb rehabilitation exoskeleton is shown in Figure 4. Notwithstanding the similarities, several distinctions between the hip and knee joints are worth noting:

(1) Dissimilarities in joint angles during movement necessitate distinct safety limit and support frame structures for each joint;
(2) While the hip joint mechanism interfaces with the human waist, the upper segment of the knee joint mechanism interfaces with the thigh bar. The knee joint mechanism contains a thigh rod connecting rod and no waist link structure;
(3) In order to improve the overall appearance of the support frame, the end cover and support frame have been designed with certain improvements.

Based on the analysis of the ankle joint mechanism, the ankle joint mechanism of the lower limb rehabilitation exoskeleton has the following characteristics:

(1) The ankle joint mechanism has as the freedom of dorsiflexion and plantar flexion;
(2) The ankle joint mechanism is under-actuated and needs to have elastic elements to self-align;
(3) An elastic energy storage deformation and vibration damping mechanism is installed at the sole of the foot to increase the overall comfort of the mechanism.

**Figure 5:**
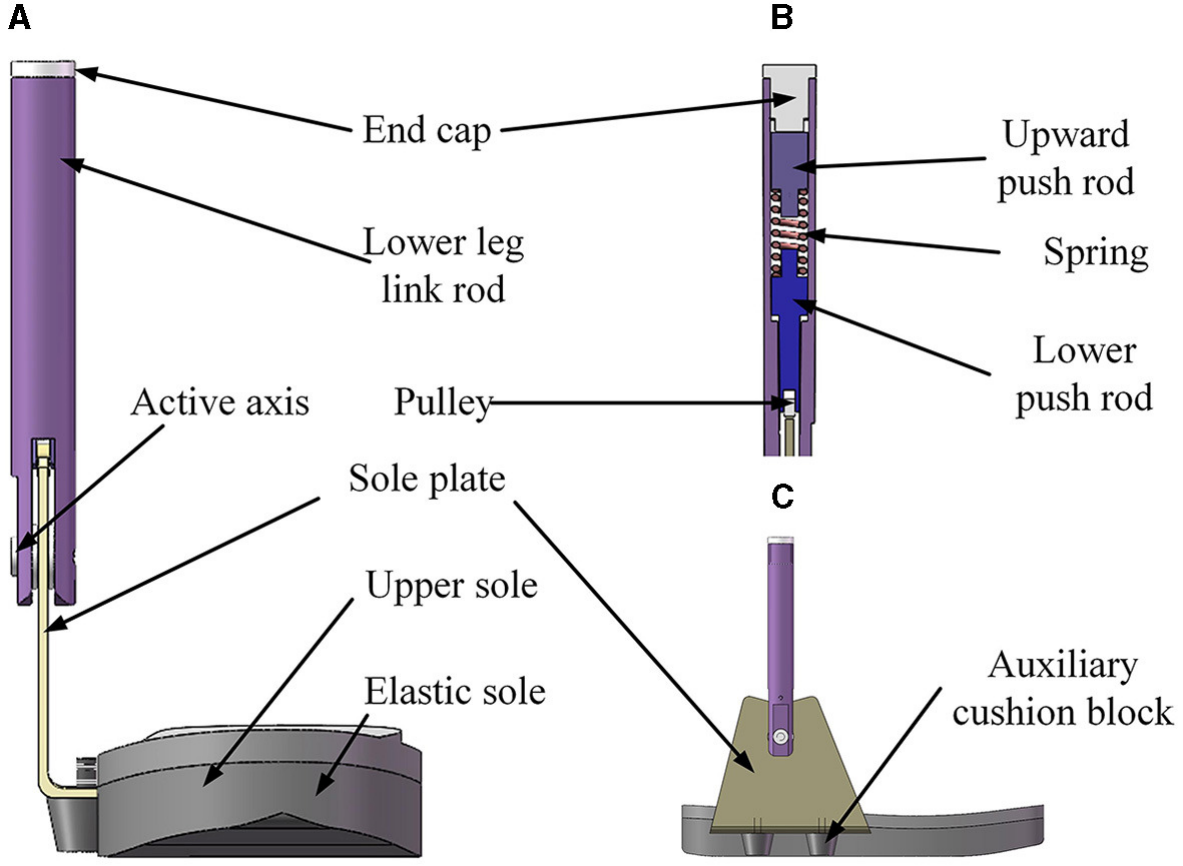
Ankle joint mechanism of lower limb rehabilitation exoskeleton. **(A)** Front view; **(B)** section view; **(C)** side view.

The ankle joint mechanism of the lower limb rehabilitation exoskeleton is shown in Figure 5. The designed ankle joint mechanism encompasses several components: an end cap, a lower leg link rod, an upward push rod, a spring, a lower push rod, a pulley, a sole plate, an active axis, an upper sole, an elastic sole, and an auxiliary cushion block. Its movement principle is: when the sole of the wearer's foot is subjected to external force, the sole plate is driven by the sole to rotate around the axis, and the lower push rod is driven to move upward to compress the spring; when the external force disappears or decreases, the spring pushes the lower push rod downward, driving the sole plate to rotate around its axis, realizing that the ankle joint is in an under-driven form and has the function of autonomous dorsiflexion and plantar flexion freedom.

### 2.3 Motion and force analysis

The active drive of the ankle joint mechanism in the lower limb exoskeleton robot offers advantages in gait control and walking stability. However, it often necessitates additional drive sources, leading to increased complexity and bulkiness in the ankle joint structure. The focus of this study lies in catering to patients with lower limb paralysis. The ankle joint mechanism equipped with functionalities of support, dorsiflexion, and plantarflexion can suffice for daily life activities, with the capacity for rotation that can autonomously return to a supporting position in an unstressed state. Based on the analysis of ankle joint motion mechanism, the sagittal plane of the ankle joint mechanism exhibits a dorsiflexion or plantarflexion range spanning from +20° to −20°, with the joint's torsional moment ranging between +3 to −3 Nm. Given these prerequisites, the ankle joint mechanism in this study is designed as an underdriven, elastic, and flexible structure.

**Figure 6:**
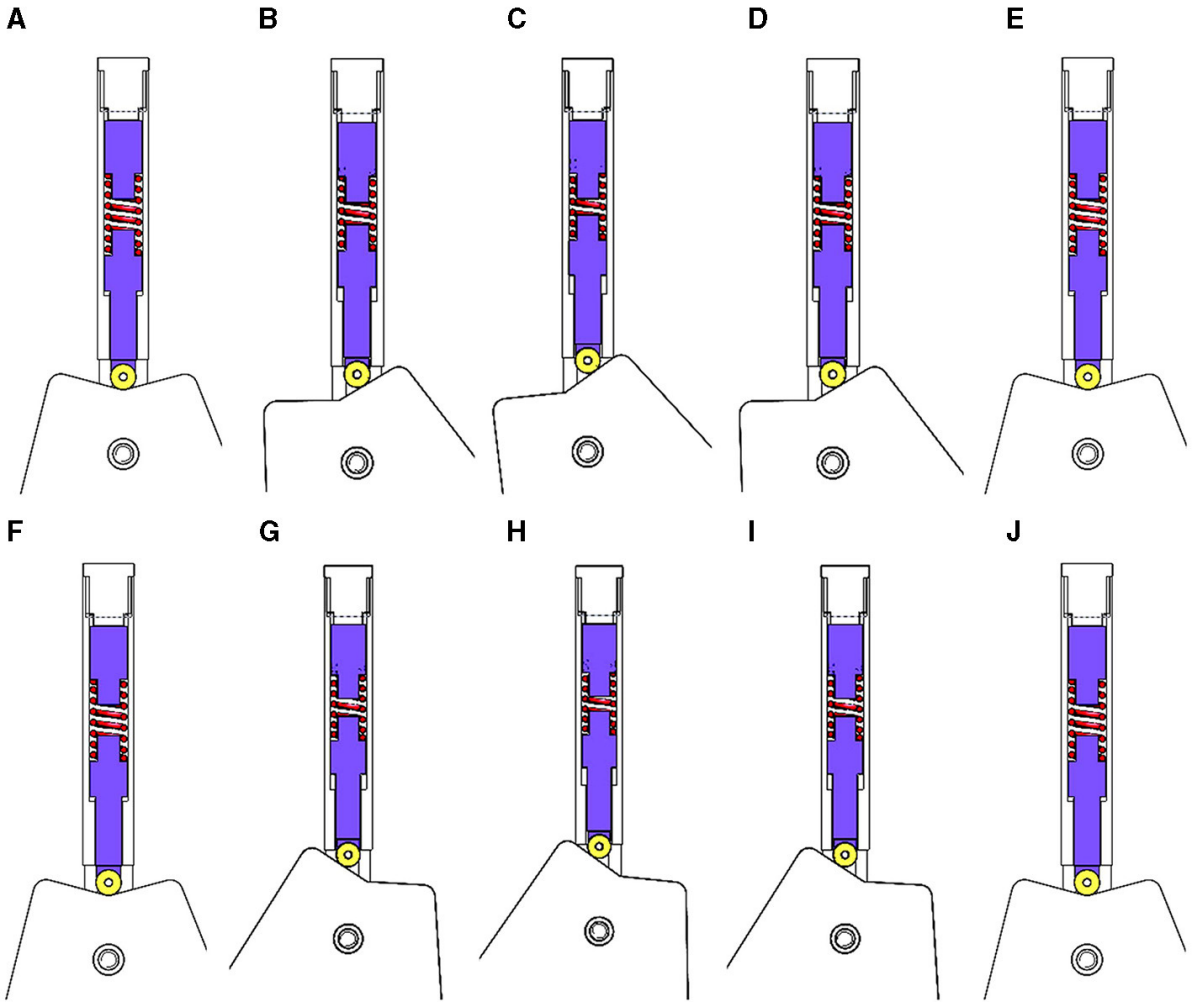
Posture changes of ankle joint mechanism. **(A–E)** Ankle dorsiflexion movement; **(F–J)** ankle plantar flexion movement.

The ankle joint mechanism of the lower limb rehabilitation exoskeleton robot has a motion range of dorsiflexion and plantar flexion in the sagittal plane of +20° to −20°. As the angle of rotation increases, a larger joint torsional moment becomes imperative. When the rotation angle reaches 20°, the joint torsional moment encompasses an approximate range of 3 Nm. The design process entails comprehensive calibration, precise determination of the distance change of the rotation center, appropriate spring selection, and pulley trajectory design. Figure 6 illustrates the posture change diagram of the ankle joint mechanism in the lower limb rehabilitation exoskeleton, depicting its movement from the support position to the maximum dorsiflexion, back to the support position, and subsequently to the maximum plantarflexion before returning to the support position.

**Figure 7:**
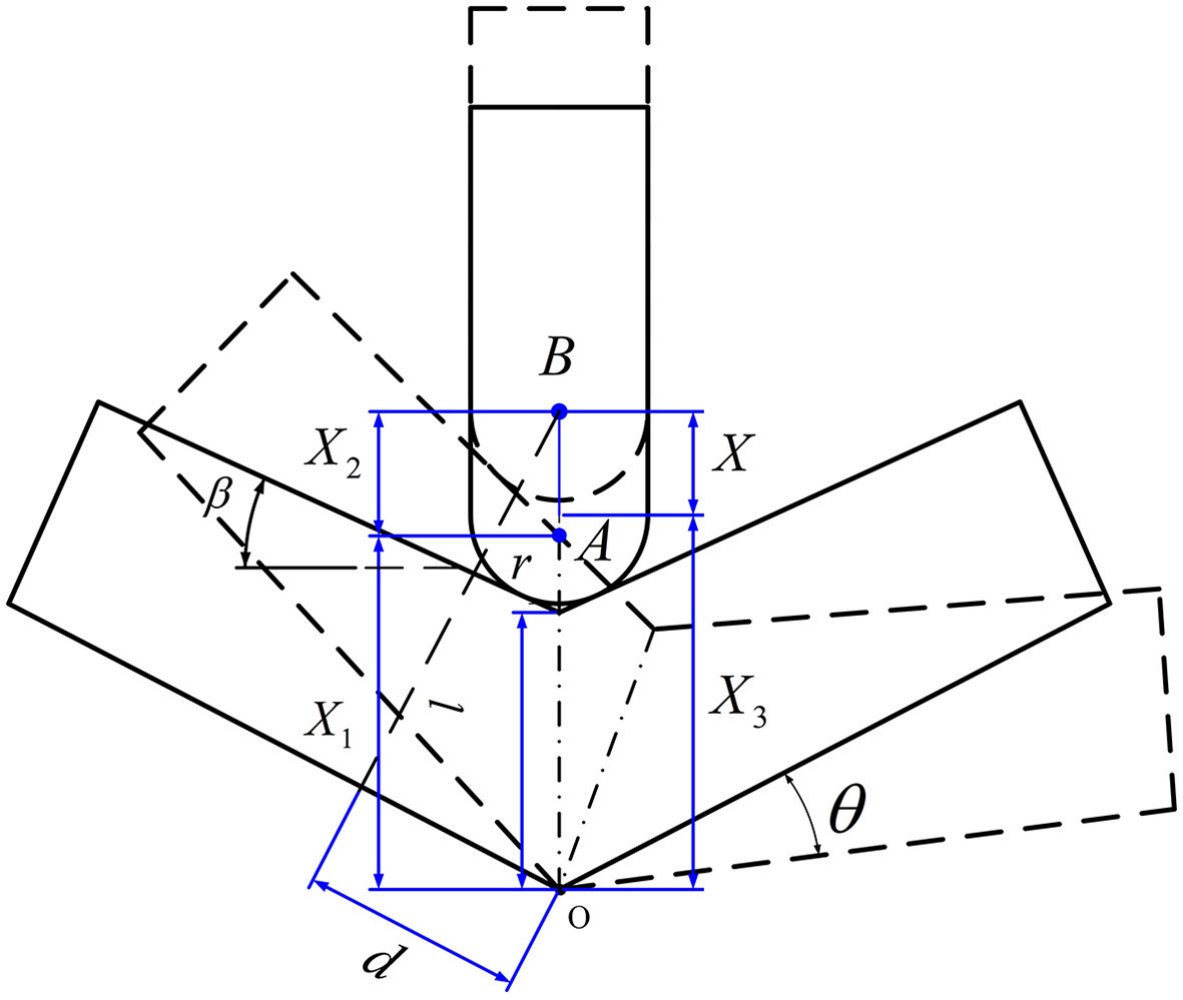
Ankle joint motion analysis of the lower limb rehabilitation exoskeleton.

In addressing the aforementioned requirements, modeling and analysis are performed, as illustrated in Figure 7. The diagram depicts the mechanism's motion, with the black solid line representing the initial state (equilibrium support position), and the black dashed line denoting an arbitrary state of the mechanism's movement. In the schematic, Point O represents the center of rotation, Point A represents the initial center of the pulley, Point B represents the center of the pulley in an arbitrary state, θ represents the angle of rotation, *X* represents the value corresponding to the change in height of actuator (from Point A to Point B), *r* represents the radius of the pulley, and *l* represents the distance from the lowest point on the upper part of the sole plate to the center of rotation. Other analysis parameters include: spring stiffness *k*, spring pressure *F*, joint moment of force *M*, torque effective distance *d*. *X*_1_ represents the distance from an state trajectory point (intersection of trajectory diagonal and vertical central axis) to the center of rotation, *X*_2_ represents the distance from the arbitrary state trajectory point to the center of the pulley, *X*_3_ represents the distance from Point O to Point A, and β represents the angle between the initial position of the inclined plane and the horizontal line.

According to geometric relations, we can get:


(1) $$\begin{array}{c} \frac{l}{\sin\left(\pi /2-\theta -\beta \right)}=X_{1}/\sin\left(\pi /2+\beta \right) \end{array}$$

(2) $$\begin{array}{c} X_{2}=r/\cos\left(\beta +\theta \right) \end{array}$$

(3) $$\begin{array}{c} X_{3}=l+r/\cos\beta \end{array}$$

(4) $$\begin{array}{c} \text{X}=X_{1}+X_{2}-X_{3} \end{array}$$

According to Equations (1)–(4), *X* can be expressed as follows:


(5) $$\begin{array}{c} X=l\cdot \frac{\sin\left(\pi /2+\beta \right)}{\cos\left(\beta +\theta \right)}+\frac{r}{\cos\left(\beta +\theta \right)}-l-\frac{r}{\cos\beta } \end{array}$$

The torque effective distance *d* can be get:


(6) $$\begin{array}{c} d=\left(X_{1}+X_{2}\right)\cdot \sin\left(\beta +\theta \right) \end{array}$$

The spring force *F* and moment of force *M* are as follows:


(7) $$\begin{array}{c} F=K\cdot \text{X} \end{array}$$

(8) $$\begin{array}{c} M=F\cdot d \end{array}$$

According to Equations (5)–(8), the effective joint moment *M* is calculated as:


(9) $$\begin{array}{c} M=k\cdot \left(l\cdot \frac{\sin\left(\pi /2+\beta \right)}{\cos\left(\beta +\theta \right)}+\frac{r}{\cos\left(\beta +\theta \right)}-l-\frac{r}{\cos\beta }\right)\\ \cdot \left(X_{1}+X_{2}\right)\cdot \sin\left(\beta +\theta \right) \end{array}$$

Calculated according to Equation (9), the moment *M* and angle θ that meet the design requirements can be obtained.

Due to the presence of friction in the mechanism, the minimum pressure angle is firstly verified to prevent the mechanism from self-locking phenomenon. Assume the following parameters: the pressure angle α, the minimum pressure angle α_min_, and friction coefficient μ (value is 0.2), we can get:


(10) $$\begin{array}{c} F_{f}=F\cdot \cos\alpha \cdot \mu \end{array}$$

(11) $$\begin{array}{c} F_{t}=F\cdot \sin\alpha \end{array}$$

The force of friction and positive pressure can be calculated according to Equations (10) and (11). At the instance when force of friction *F*_*f*_ equals positive pressure *F*_*t*_, it corresponds to the position where the minimum pressure angle, α_min_ is 11°. According to the above calculation, the pressure angle of this structure is β+θ. Since 0° ≤ θ ≤ 20°, β ≥ 11° can ensure that the mechanism will not self-lock.

In the elastic component design stage, a compression spring with ends on both sides was chosen. In alignment with the ankle joint mechanism dimensions derived from the aforementioned calculations, an appropriate size and stiffness for the spring were determined. Additionally, considerations pertaining to installation dimensions and other related factors were also taken into account. After repeated verification and design, the specific performance parameters of the compression spring are presented in Table 3.

**Table 3:** Performance parameters of compression springs.

**Material**	**SWP-B(2.5)GB/T**	**Coiled way**	**Dextrorotation**
Diameter	14.5 mm	Spin ratio	4.803
Free length	32 mm	Spring unit weight	12.13 g/pcs
Active coils	6.3	Pitch	4.28
Telomorphism	Rounded and smoothed	Rigidity	37 N/mm

**Figure 8:**
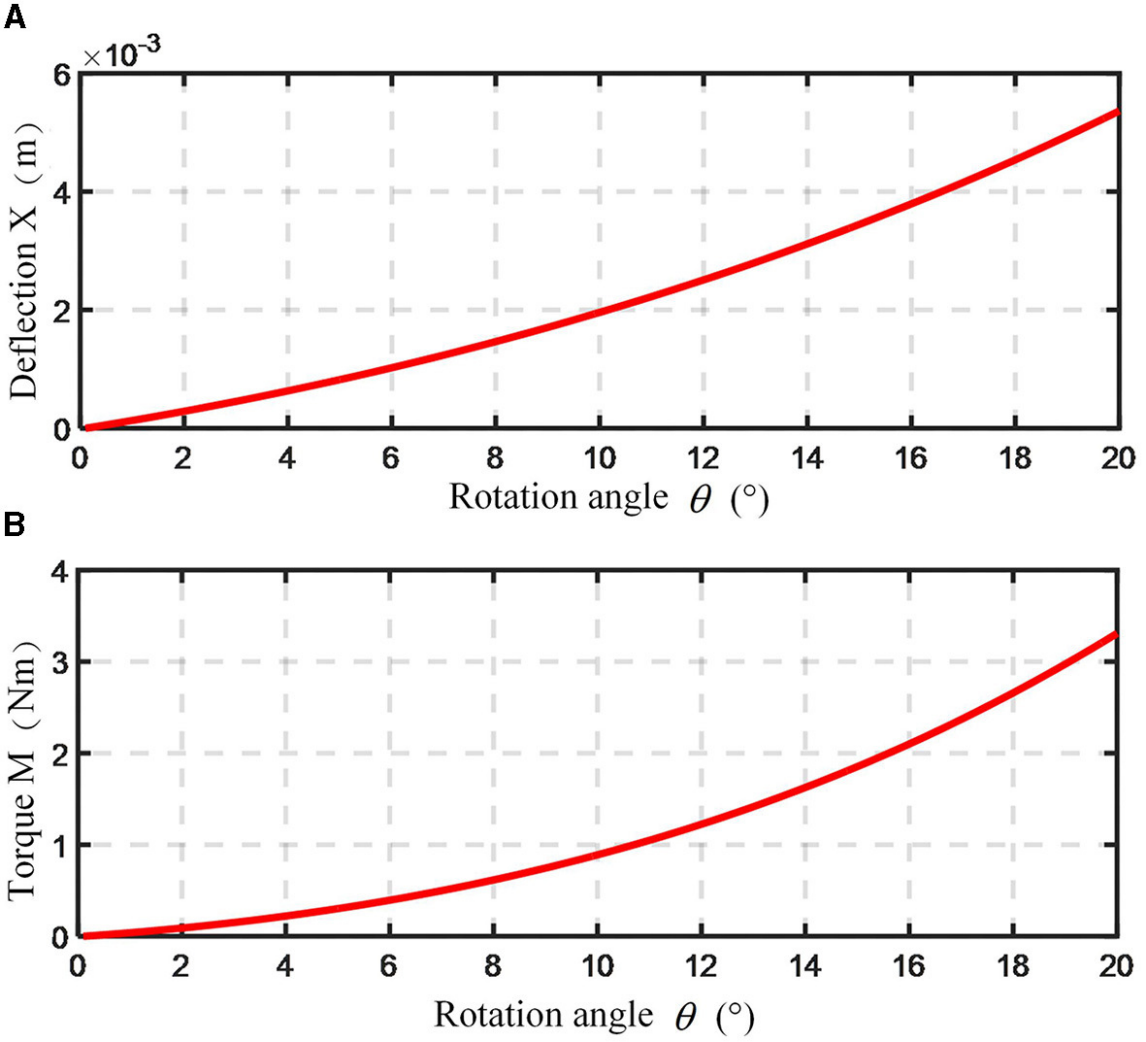
Variation curve of ankle spring deformation and moment. **(A)** Relationship between spring deformation and ankle joint angle; **(B)** relationship between moment and ankle joint angle.

Upon analysis, the spring stiffness *k* is 37 N/mm. According to the design requirements, when θ is 20°, *M* approximates 3 Nm, it is concluded that β is 15° and *l* is 25 mm. Relationships between ankle angle θ and spring deformation *X*, as well as ankle angle θ and torque *M* are shown in Figure 8. It becomes evident that as the rotation angle θ escalates, the torque *M* also experiences an increase, reaching 3.1 N·m when θ is 20°.

## 3 Exoskeleton robot control system

### 3.1 Robot system composition

The lower limb exoskeleton primarily serves patients afflicted with lower limb motor dysfunction, essentially functioning as a mechatronic device that aids in rehabilitation training and facilitates the restoration of upright walking capabilities (Baud et al., 2021). The hardware design of the lower limb exoskeleton control system ought to adhere to fundamental principles:

(1) The primary controller must possess adequate peripheral interfaces capable of receiving diverse sensor signals integrated within the lower limb exoskeleton. Moreover, it is imperative for the human-machine interaction signals and underlying algorithms to exhibit expedited response times, enabling seamless adaptation to varying motion patterns and expeditious computation of control inputs for joint motors;
(2) Operating as a rehabilitation robot, the lower limb exoskeleton necessitates real-time, direct interaction with the patient, along with the capacity to document rehabilitation training data on storage devices or visualize it via monitors;
(3) Given its role as an assistive robot for patients with lower limb motor impairments undergoing gait training, ensuring safety, reliability, and stability is of paramount importance in the hardware design of the lower limb exoskeleton control system.

**Figure 9:**
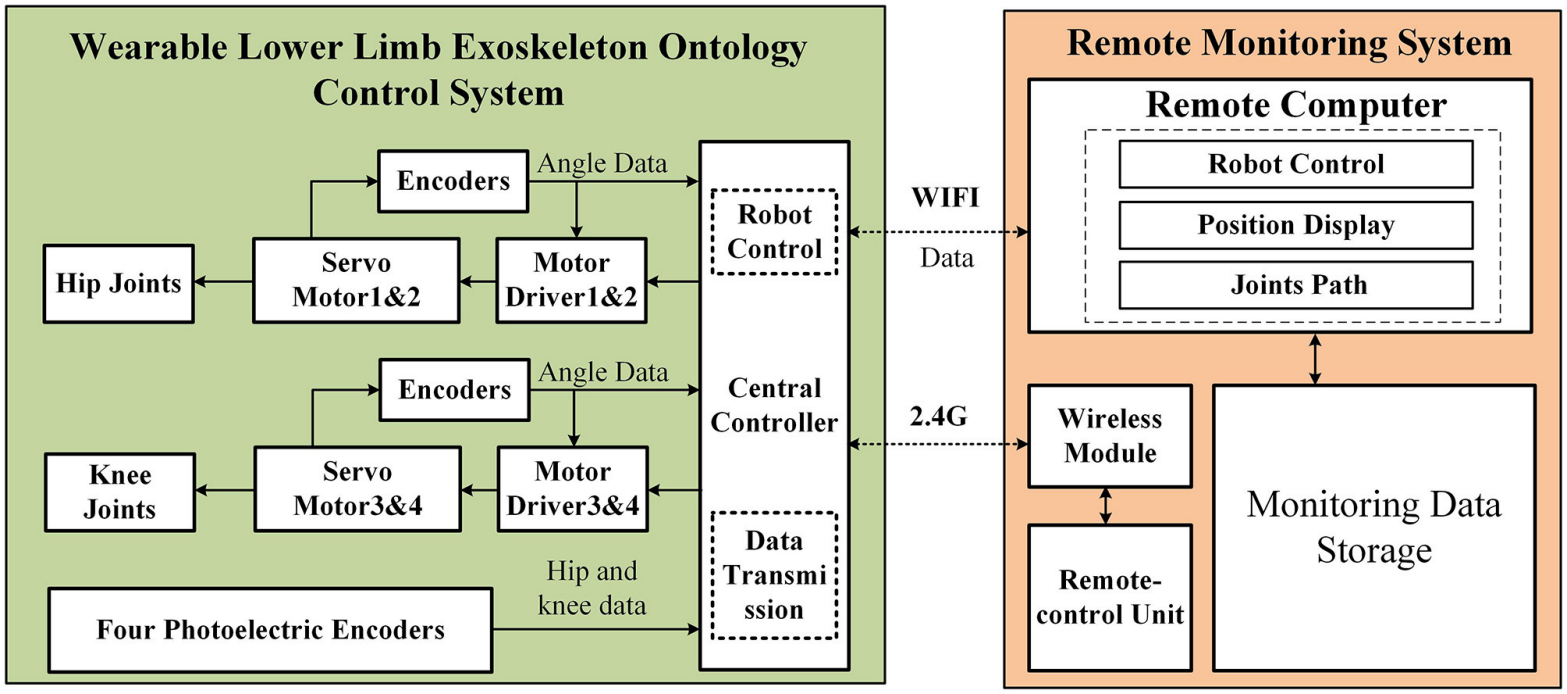
Control system hardware composition of the lower limb rehabilitation exoskeleton.

The control system hardware composition of the lower limb rehabilitation exoskeleton is shown in Figure 9. The hardware components of the lower limb rehabilitation exoskeleton consist of two subsystems: the wearable lower limb exoskeleton ontology control system and the remote monitoring system. The wearable lower limb exoskeleton ontology control system encompasses elements such as the central controller, motor drivers, servo motors, encoders, and photoelectric encoders. On the other hand, the remote monitoring system comprises a remote control unit and a remote computer. The wearer can operate the remote control unit to wirelessly transmit signals to the central controller, which subsequently issues corresponding control directives to the motor drivers. The motor drivers then activate the motors, initiating movement in the hip and knee joint mechanisms. The encoders are responsible for acquiring data and providing feedback to the central controller, facilitating closed-loop control. Concurrently, the central controller relays data in real-time to the remote computer, which is tasked with storing and exhibiting trajectory and joint information data.

### 3.2 Robot control method

**Figure 10:**
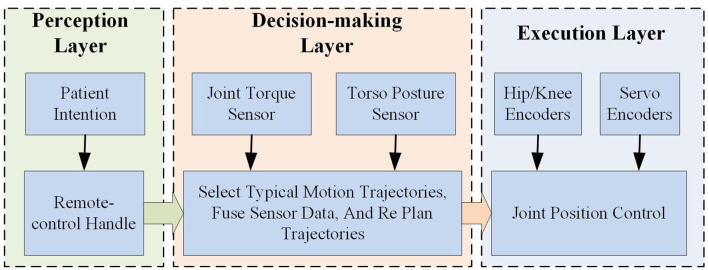
Control layer logic diagram of the lower limb rehabilitation exoskeleton.

The control framework of the lower limb rehabilitation exoskeleton consists of three layers: the perception layer, the decision-making layer, and the execution layer, which collectively control the perception, decision-making, and execution faculties of the exoskeleton robot. Figure 10 illustrates the logical architecture of the lower limb rehabilitation exoskeleton control framework. Patients manipulate the exoskeleton to operate in various modes, including standing up, sitting down, continuous walking, and climbing or descending stairs, through buttons positioned on the right crutch-mounted segment. Subsequently, the output from the perception layer conveys the designated trajectory for the exoskeleton to the decision-making layer. The decision-making layer utilizes an optimized typical joint trajectory as the foundational trajectory for trajectory tracking. Concurrently, this layer acquires human-computer interaction data via sensors, further refines and adjusts the trajectory being tracked by the exoskeleton, and ultimately relays the specific joint motion state information pertaining to the present moment to the execution layer. The execution layer processes the feedback signals from the sensors and the trajectory devised by the upper layer, achieving high-precision regulation of the joint motion state through a servo control driver.

**Figure 11:**
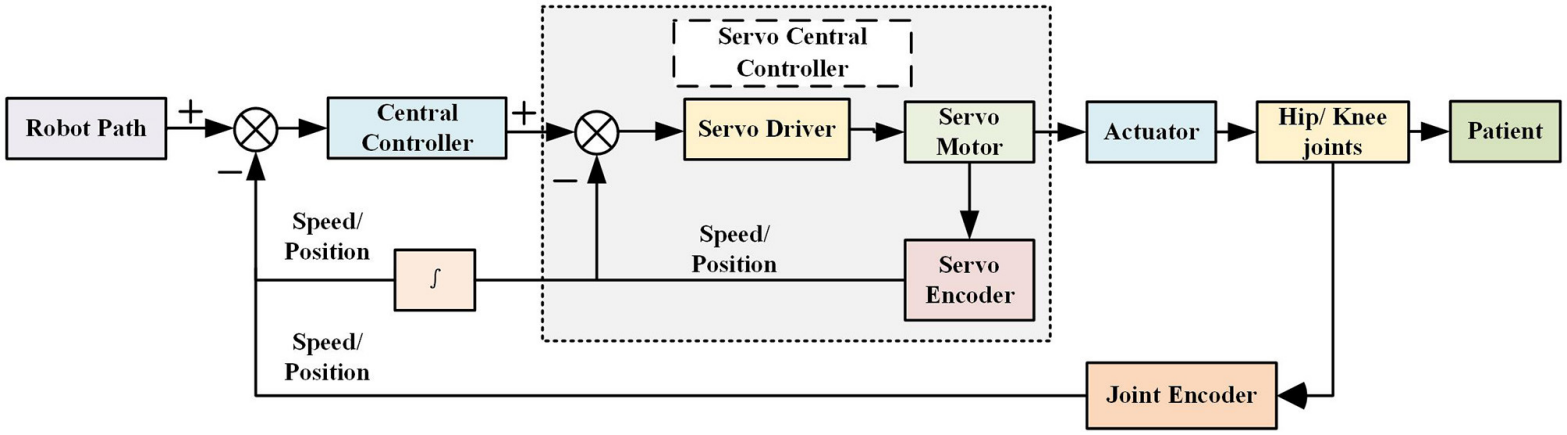
Control method of the lower extremity rehabilitation exoskeleton.

A dual closed-loop PID control strategy tailored to joint motion is devised based on the prerequisites of early rehabilitation training. The detailed block diagram of this control strategy is depicted in Figure 11, facilitating the precise tracking of exoskeleton joints along the desired trajectory. Utilizing this ideal trajectory as a foundation, a method for reproducible trajectory planning through the quantification of human-machine rejection was proposed. Additionally, an innovative human-machine gait joint moment cycle learning algorithm is adopted to compute the degree of human-machine rejection. To refine the ideal trajectory, a fuzzy controller is implemented, and its efficacy is substantiated through extensive human-computer experiments, enabling the attainment of trajectory reprogramming and tracking. To ensure the system's expedited response to perturbations, a fuzzy PID controller is integrated into the closed-loop joint position, thereby optimizing the system's overall performance.

## 4 Experiments and tests

Given the specificity that the lower limb exoskeleton will be directly applied to human lower limbs, the significance of its safety, stability, and reliability is particularly emphasized. To ensure the safety of the users, some fundamental experiments on the lower limb rehabilitation exoskeleton were performed: robotic motion response experiments and robotic trajectory tracking experiments. These experiments aim to assess the system's performance before progressing to experiments involving walking, standing up, and sitting down with able-bodied individuals.

### 4.1 Robot motion response experiments

The designed lower limb rehabilitation exoskeleton is mainly used to assist patients with lower limb paralysis to achieve basic daily movements. In practical control implementations, this exoskeleton is expected to exhibit swift responsiveness, attaining the desired angular positions as per control directives expeditiously. Consequently, conducting robotic motion response experiments for this lower limb rehabilitation exoskeleton becomes imperative.

**Figure 12:**
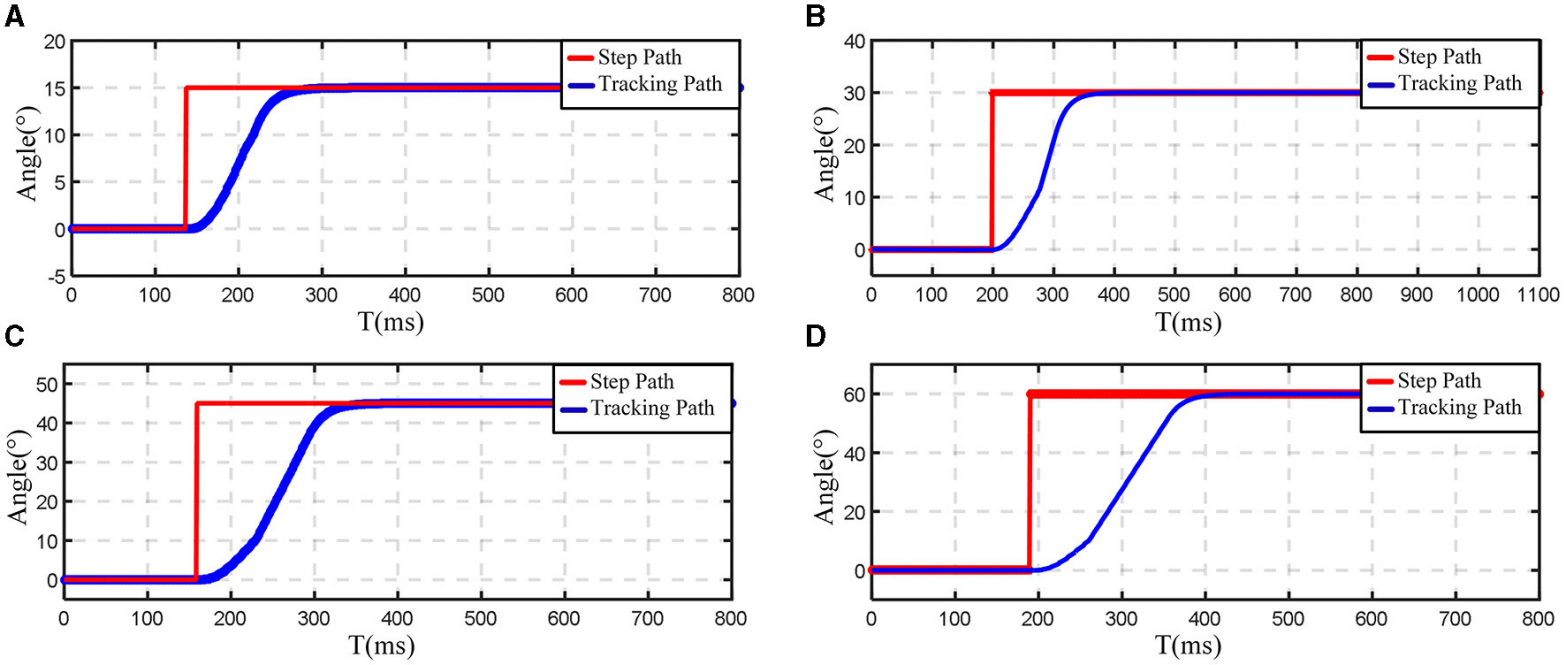
Experimental results of hip joint angular response. **(A)** 15°; **(B)** 30°; **(C)** 45°; **(D)** 60°.

Since the motion structures of the hip and knee joints are basically similar, the hip joint mechanism was selected for the robot motion response experiments. These response experiments were conducted at desired angles of 15°, 30°, 45°, and 60°. The specific experimental results are presented in Figure 12. At an angle of 15°, the adjustment time is approximately 160 ms with a lag time of about 10 ms. Similarly, at angles of 30° and 45°, the adjustment times are around 180 and 200 ms, respectively, with a consistent lag time of about 10 ms. This lag can be primarily attributed to the use of a worm gear transmission. The backlash between the forward and reverse gears introduces return errors, leading to a delay in the rotation of the lower limb rehabilitation exoskeleton in the reverse direction.

Although the exoskeleton system's mechanical characteristics introduce a certain degree of lag in response, this lag time is practically negligible relative to the entire gait cycle. The experimental results demonstrated that the lower limb rehabilitation exoskeleton system's joint angle response speed could satisfy the practical control requirements for lower limb rehabilitation exoskeletons. The exoskeleton system performance meets the expectation for assisting patients with lower limb paralysis in executing daily movements.

### 4.2 Robot trajectory tracking experiments

The control strategy employed by the lower limb rehabilitation exoskeleton relies on passive control, which underscores the significance of the mechanism's trajectory tracking proficiency. The primary technical metric for the lower limb rehabilitation exoskeleton is its ability to precisely track the desired trajectory when operating in an unloaded state. In order to evaluate the trajectory tracking performance of the robot, during the experiment, the optimized motion curve was used as input, representing the expected gait trajectory. Meanwhile, the encoder captures the actual angular positions of the hip and knee joints of the exoskeleton, which are treated as the output or tracking trajectory. By analyzing the real-time discrepancies between the input and output trajectories, the trajectory tracking features of the exoskeleton were validated, thus ensuring its efficacy in assisting lower limb rehabilitation.

**Figure 13:**
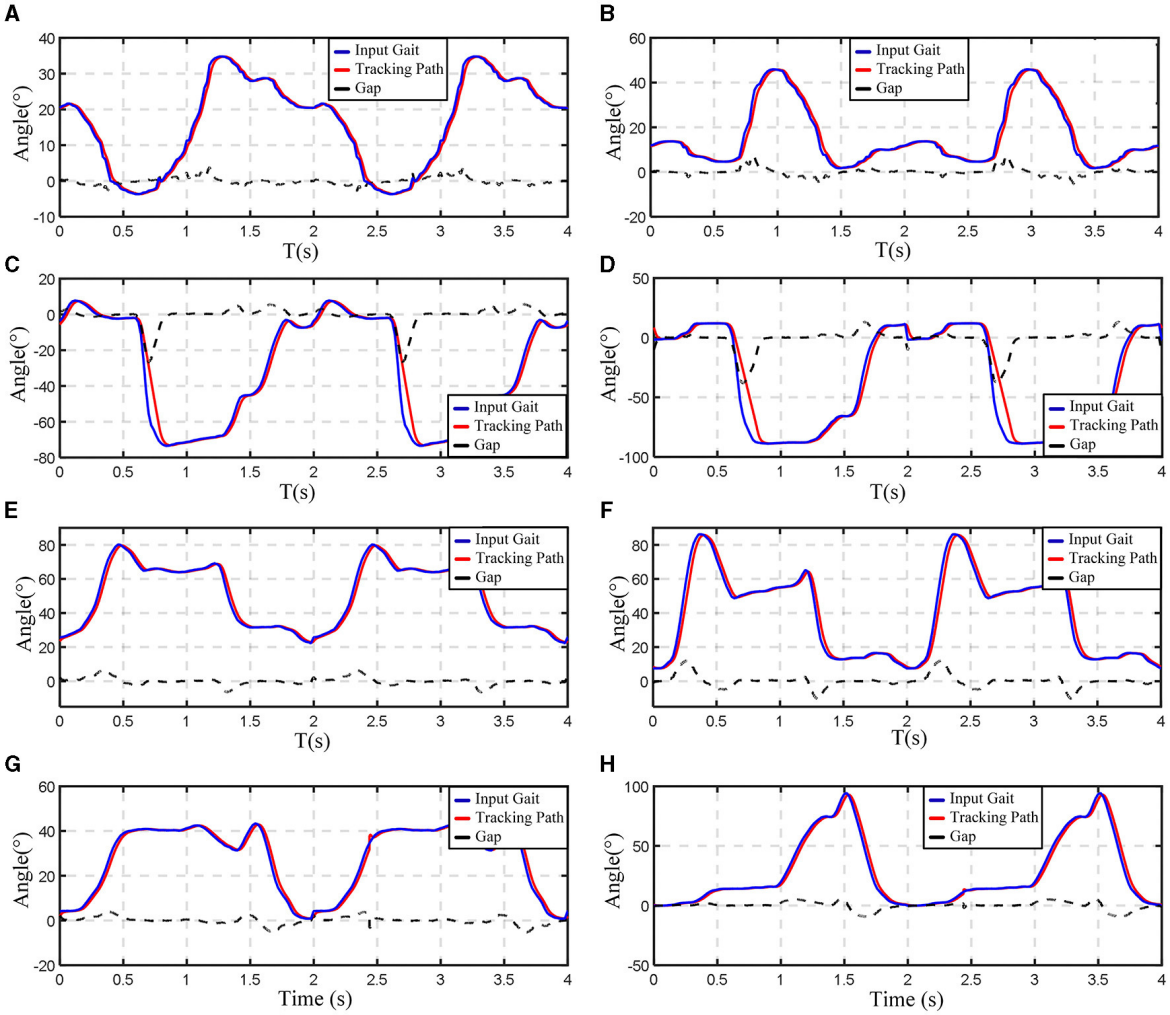
Experimental results of trajectory tracking. **(A)** Hip joint angle change while walking on crutches; **(B)** knee joint angle change while walking on crutches; **(C)** hip joint angle change while standing up and sitting down; **(D)** knee joint angle change while standing up and sitting down; **(E)** hip joint angle change while walking down the stairs; **(F)** knee joint angle change while walking down the stairs; **(G)** hip joint angle change while walking up the stairs; **(H)** knee joint angle change while walking up the stairs.

In the initial crutch walking trajectory tracking experiments, the optimized crutch walking joint curves were utilized as inputs and fed into the controller. The resulting output curves were collected. The trajectory tracking curves for both the hip and knee joints during crutch walking are presented in Figures 13A, B. The gait trajectory is represented by the blue line, the real-time tracking curve of the mechanism is shown in red, and the correction amount (or gap) is depicted by the black dotted line. It is evident that, within a gait cycle, the experimental results for crutch walking trajectory tracking are highly satisfactory. The tracking errors for both the hip and knee joints are consistently maintained within a range of ±3°, with the majority of the errors falling within ±1°. The tracking accuracy can meet the functional requirements of the exoskeleton.

Following the successful completion of the crutch walking trajectory tracking experiments, additional experiments were conducted to evaluate the exoskeleton's performance during activities such as standing up/sitting down with crutches and ascending/descending stairs with crutches. The results of these experiments are displayed in Figures 13C–H. The experimental results indicated that the actual tracking trajectory trended closely with the gait trajectories. The trajectory tracking errors are within a small range of ±5°, demonstrating excellent trajectory tracking capabilities for this unloaded lower limb rehabilitation exoskeleton.

From the figure, it can also be found that the tracking trajectory relative to the gait trajectory has a certain phase lag, which is mainly caused by two reasons: on the one hand, due to the worm gear has the return error characteristics; on the other hand, due to the motor needs a certain response time. In the experiments, the vast majority of the lag time is very small, basically in the range of 5 ms, and does not affect the overall performance of the mechanism.

The tracking curve and gait curve maximum error appeared in the trajectory tracking experiments on crutches standing up, and the instantaneous maximum error of 20°. By analyzing the gait trajectory, it can be found that the acceleration at the moment of standing up was too large, which was caused by the sudden change in speed (the curve was close to 90°). Although the error is large at this time, the tracking trajectory is always the same as the gait trajectory. There is a certain phase deviation and curvature difference, and the maximum deviation is <100 ms, which can meet the performance of the lower limb rehabilitation exoskeleton standing action.

There is a certain lag between the tracking trajectory and the gait trajectory, but its trend is always consistent and the error is within an acceptable range. The experimental results indicated that the lower limb rehabilitation exoskeleton had an excellent trajectory tracking performance under unloaded condition, and could meet the actual motion requirements.

### 4.3 Human-machine wearing tests

The safety, stability, and reliability of the developed lower limb rehabilitation exoskeleton mechanism have been verified through series of experiments. Tests involving normal humans wearing the exoskeleton were carried out, encompassing a range of activities such as walking, standing up, and sitting down.

In the preliminary experiments, gait curves and moment curves for the assisted walking, standing up, and sitting down processes of the lower limb exoskeleton were obtained. These curves were derived by measuring joint angle values and effective moment values in the sagittal plane of the active joints. The characteristics of these curves are used to verify the reliability and validity of the lower limb exoskeleton's assisted walking functionality. Additionally, subjective evaluations provided by the experimental subjects were utilized to assess the performance of the exoskeleton mechanism in assisting human movement.

**Figure 14:**
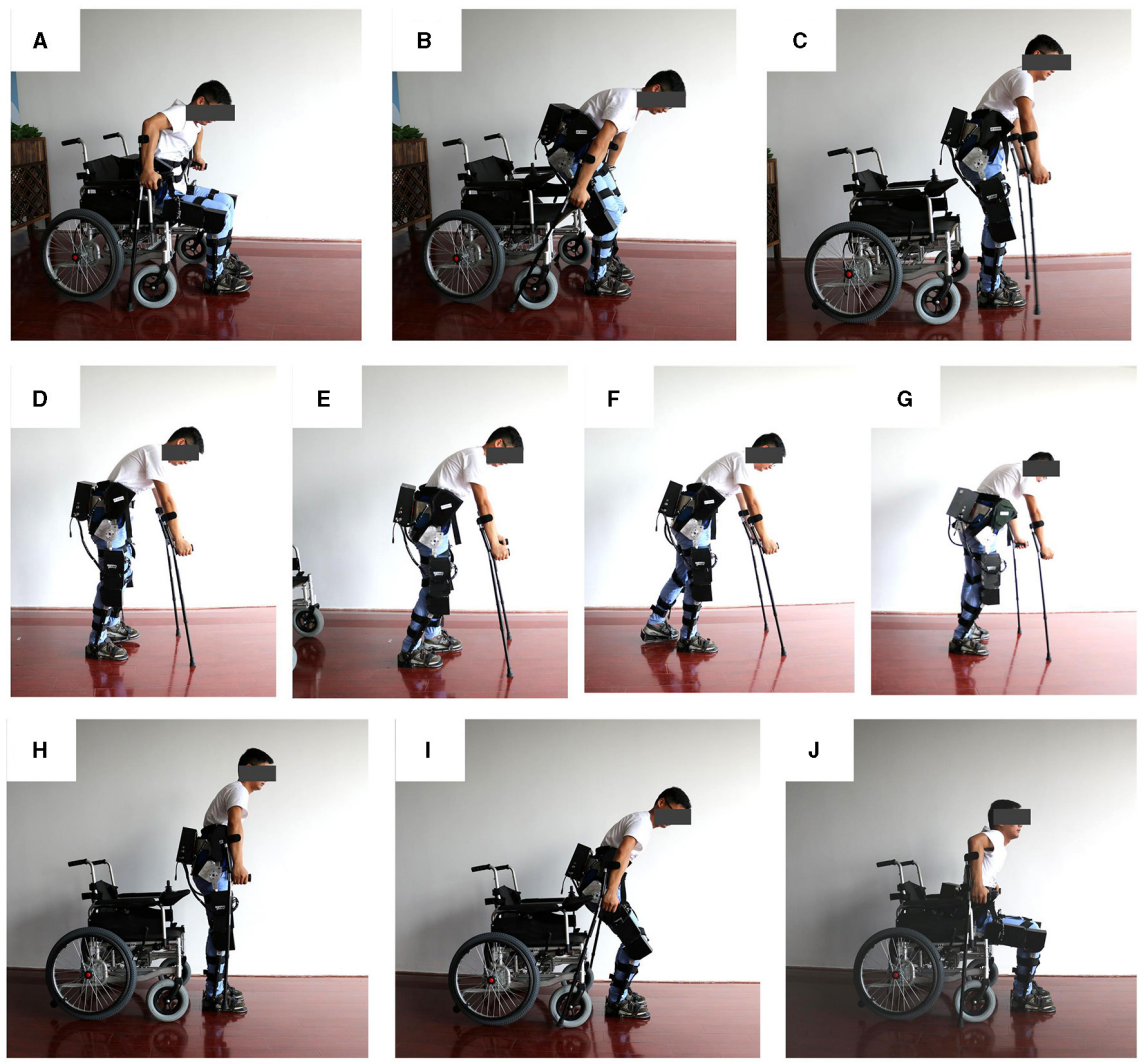
Test process of the lower limb rehabilitation exoskeleton robot worn by normal people. **(A–C)** Standing up test; **(D–G)** walking test; **(H–J)** sitting down test.

During the test procedure, three healthy and normal experimental subjects were selected. The first subject was 24 years old, weighed 65 kg, and stood 174 cm tall. The second subject was 25 years old, weighed 62 kg, and measured 168 cm in height. The third subject was 50 years old, weighed 65 kg, and was 176 cm tall. The tests primarily consisted of standing up, walking, and sitting down activities. These subjects wore the lower limb rehabilitation exoskeleton, which was placed in a wheelchair. They then performed the designated tasks while being supported by the exoskeleton and crutches. Specifically, the subjects stood up with assistance from the crutches and the exoskeleton, walked in a straight line for ~10 meters while being supported by the crutches and exoskeleton, and finally sat down with the aid of the crutches and exoskeleton. Figure 14 shows the process of standing up, walking and sitting down in a normal human wearing the lower limb rehabilitation exoskeleton.

**Figure 15:**
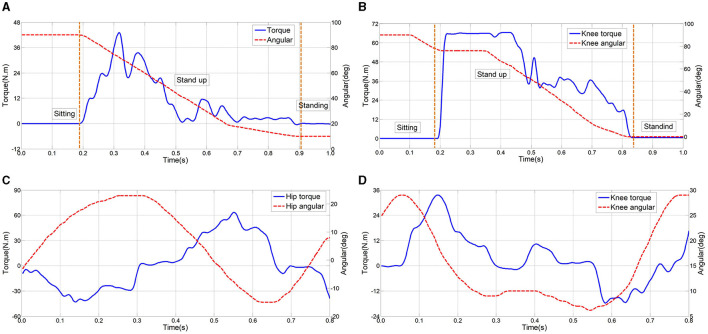
Test results of normal human wearing exoskeletons. **(A, B)** During stand-up stage; **(C, D)** during walking stage.

Through multiple tests, active joint angle and moment curves during motion were acquired. For analysis, a set of active joint angle and moment curves during the stand-up phase and another set within one gait cycle were selected as performance characteristic curves. Test curves of a normally-abled individual wearing an exoskeleton during stand-up are shown in Figures 15A, B, encompassing hip and knee joint angle and moment curves. The blue line represents actual joint moment curves, while the red line denotes actual joint angle curves, with the yellow-shaded area indicating the stand-up process. The joint angle curve trends align with the body's rising motion, with peak effective moments reaching 44 Nm for the hip and 68 Nm for the knee. The effective assistance can be provided by the lower limb rehabilitation exoskeleton during stand-up, enabling subjects to complete the action with support, thereby validating the exoskeleton's reliability and effectiveness in facilitating the stand-up process.

Test results of normal human walking wearing the lower limb rehabilitation exoskeleton are shown in Figures 15C, D. The exoskeleton gait aligns seamlessly with human walking patterns, exhibiting smooth, noise-free curves, thereby reinforcing the mechanism's reliability and stability. According the moment curves of both hip and knee joints, in a single gait cycle, the maximum effective moment for the hip joint reaches 65 and 34 Nm for the knee joint. These variations mirror typical gait patterns observed in healthy individuals. Synchronization between moment and angle curves, evident from their respective values and trends, indicates the effective assistance provided by the lower limb rehabilitation exoskeleton to subjects during walking, thus verifying the exoskeleton's reliability and efficacy.

In the human-machine wearing tests, the peak torque value is smaller than the peak torque value when normal people walk. The main reasons include: (1) when the normal person wears the exoskeleton, the exoskeleton can provide gait torque, the human body may also provide some effective torque; (2) Using crutches to assist walking can appropriately reduce the joint torque required for actual walking.

Through interviews conducted with the three experimental subjects, users generally indicated that they could distinctly sense the assisted movement provided by the exoskeleton during walking, standing up, and sitting down. This was particularly evident in scenarios where, while walking, the experimental subjects consciously relinquished control of their lower limbs, allowing the exoskeleton to drive their lower limb movements. With the aiding effect of their upper limbs and crutches, they could adeptly execute the prescribed gait patterns. Moreover, during the processes of standing up and sitting down, even though it is challenging to assert that the human body did not contribute any assisting force, the experimental subjects could unmistakably perceive the assisted thrust generated by the exoskeleton.

Through the aforementioned experiments and subsequent analysis, it becomes evident that the lower limb rehabilitation exoskeleton offers substantial assistance to the experimental subjects in facilitating normal activities such as standing up and walking. The lower limb rehabilitation exoskeleton robot effectively aids the experimental subjects in accomplishing a wide range of movements, thereby validating the strong reliability, efficacy, and stability of the lower limb rehabilitation exoskeleton presented in this study.

## 5 Conclusion

In this paper, we propose a new exoskeleton robot system for lower limb rehabilitation. According to the design requirements, the mechanical structural design and force analysis of the lower limb rehabilitation exoskeleton are carried out. The structural components of the lower limb rehabilitation exoskeleton mainly include: the backpack mechanism, the hip joint mechanism, the knee joint mechanism and the ankle joint mechanism. The exoskeleton control system executes actions through a variety of hardware, such as sensors and drive motors, and can realize closed-loop position control and trajectory planning control of each exoskeleton joint mechanism. A series of performance experiments and wearing tests were conducted on the designed lower limb rehabilitation exoskeleton device. In the robot angle response experiment, four response angles were verified, including: 15°, 30°, 45°, and 60°. The experimental results indicated that the exoskeleton joints have fast response characteristics. Through testing typical movements such as walking, standing up, and going down and up and down stairs, the robot trajectory tracking experiments verified the excellent trajectory tracking characteristics of the lower limb rehabilitation exoskeleton, with a maximum tracking error of ±5°. In addition, multiple sets of wearing tests were performed to test the assistive effect of the lower limb rehabilitation exoskeleton during walking, standing up and sitting down to verify the reliability, safety, effectiveness and stability of the mechanism. This exoskeleton robotic system helps patients perform daily movements. The test results indicated that the exoskeleton robot has good reliability and safety. This exoskeleton robotic system is conducive to performing some daily movements and sports of paralyzed patients.

In future work, we will further verify the robot wearable application experiments in more complex daily life scenarios, the control methods will be optimized by combining electroencephalogram signals and other methods. Combined with clinical needs, the patient's status will be analyzed to enhance the effect of intelligent robots in the patient's rehabilitation process.

## Data availability statement

The original contributions presented in the study are included in the article/supplementary material, further inquiries can be directed to the corresponding authors.

## Ethics statement

Ethical approval was not required for the study involving humans in accordance with the local legislation and institutional requirements. Written informed consent to participate in this study was not required from the participants or the participants' legal guardians/next of kin in accordance with the national legislation and the institutional requirements. Written informed consent was obtained from the individual(s) for the publication of any potentially identifiable images or data included in this article.

## Author contributions

ZZ: Investigation, Methodology, Writing – original draft. LL: Data curation, Methodology, Writing – review & editing. WZ: Formal analysis, Validation, Writing – review & editing. CJ: Formal analysis, Validation, Writing – review & editing. XW: Conceptualization, Funding acquisition, Supervision, Writing – review & editing. JL: Conceptualization, Project administration, Supervision, Writing – review & editing, Writing – original draft.

## Footnotes

^1^[EB/OL] Rewalk. http://www.rewalk.com.

^2^[EB/OL] http://www.gaylord.org/Our-Programs/Spinal-Cord/Ekso-Bionic-Eksoskeleton.
